# [Retracted] Overexpression of TGF-β enhances the migration and invasive ability of ectopic endometrial cells via ERK/MAPK signaling pathway

**DOI:** 10.3892/etm.2025.12934

**Published:** 2025-08-04

**Authors:** Zhihong Liu, Lisha Yi, Miaomiao Du, Guifang Gong, Yali Zhu

Exp Ther Med 17:4457–4464, 2019; DOI: 10.3892/etm.2019.7522

Following the publication of this paper, it was drawn to the Editors’ attention by a concerned reader that various of the western blots in this paper were strikingly similar to data that have been published in a number of seperate articles written by different authors at different research institutes; specifically, the control western blot data shown in Fig. 2A on p. 4460 were apparently the same as the control bands shown in Fig. 4D in a paper that was already under consideration for publication in the journal *Medical Science Monitor*, and which has subsequently been retracted. In view of the fact that the abovementioned data were already under consideration for publication when this paper was received at *Experimental and Therapeutic Medicine*, the Editor has decided that this paper should be retracted from the Journal. The authors were asked for an explanation to account for these concerns, but the Editorial Office did not receive a reply. The Editor apologizes to the readership for any inconvenience caused.

